
JOURNAL INFORMATION
==============================
NLM Title Abbreviation: Tex Med J (Austin)
NLM Journal ID: 21130590R

Texas Medical Journal

ISSN: 0892-8495
Publisher: Biomedical Journal Digitization Project

ARTICLE INFORMATION
==============================
PMCID: PMC9591970
PMID: 36955022
Article version: 1
Subjects: Society Notes

The East Texas Medico=Chirurgical Association

Guinn E. E. Secretary

Print publication date: 1902 Nov
Volume: 18
Issue: 5
First page: 203
Last page: 203
License: This work is free of known copyright restrictions. For more information, please see PMC Back Issue Digitization.
License URL: https://creativecommons.org/publicdomain/mark/1.0/

==============================

Phillips' Emulsion 50% best NORWAY COD LIVER OIL minutely sub-divided, WITH WHEAT PHOSPHATES (Phillips')
Pancreatized, Palatable, Permanent, Miscible in Water, Milk, Wine, etc.

Phillips' Milk of Magnesia Mg H2 O2 (FLUID.) "THE PERFECT ANTACID."
for correcting Hyperacid conditions—local or systemic.
Vehicle for Salicylates, Iodides, Balsams, etc.

Phillips' Phospho-Muriate of Quinine, COMP.
TONIC AND RECONSTRUCTIVE.
WHEAT PHOSPHATES, WITH MURIATE OF QUININE AND STRYCHNINE.

PHILLIPS' WHEAT PHOSPHATES (ACID).
PHILLIPS' SYRUP OF WHEAT PHOSPHATES.
PHILLIPS' DIGESTIBLE COCOA.
THE CHAS. H. PHILLIPS CHEMICAL CO., NEW YORK

### The East Texas Medico-Chirurgical Association.

*Dr. F. E. Daniel, Editor Texas Medical Journal, Austin, Texas.*

DEAR DOCTOR: Owing to my absence from the State, I will not be able to get out the program of the November meeting of the East Texas Medico-Chirurgical Society in time for it to appear in the "Red-Back," so please announce that said society will meet in Palestine November 28 and 29, 1902, and an interesting program carried out. There will be some very able papers presented.

I am at the above school (Illinois School of Electro-Therapeutics, Chicago) taking special work in X-ray and electricity and feel that I am getting the worth of my money.

With best wishes, I am

Yours, etc.,
E. E. GUINN, Secretary.

### The Brazos Valley Medical Society—Announcement.

The fourteenth semi-annual meeting of the Brazos Valley Medical Association will convene at Rockdale, Texas, on the second Tuesday and Wednesday of November, 1902.
